# Cannabis-Related Diffuse Alveolar Hemorrhage in a 16-Year-Old Patient: A Case Report

**DOI:** 10.3389/fped.2019.00468

**Published:** 2019-11-14

**Authors:** Laura Bucchino, Alice Monzani, Sara Fracon, Giulia Genoni, Tiziana Cena, Simonetta Bellone

**Affiliations:** ^1^Pediatric Unit, Department of Health Sciences, University of Eastern Piedmont, Novara, Italy; ^2^Anesthesiology and Intensive Care Unit, Accident and Emergency Department, University of Eastern Piedmont, Novara, Italy; ^3^Neonatal and Pediatric Intensive Care Unit, Maggiore della Carità University Hospital, Novara, Italy

**Keywords:** diffuse alveolar hemorrhage, acute respiratory failure, pediatrics, cannabis, sevoflurane, anesthesia

## Abstract

Diffuse alveolar hemorrhage (DAH) is a clinical condition characterized by the rapid onset of dyspnea, hemoptysis and acute respiratory failure (ARF). It is commonly caused by autoimmune systemic vasculitis, pulmonary infections, drugs and tumors. Here, we report a case of DAH caused by frequent cannabis smoking. A 16-year old boy presented with hemoptysis, dyspnea and ARF soon after laparoscopic surgery for varicocele in general anesthesia. The suspected diagnosis of DAH emerged from the initial chest radiography, and it was then confirmed by CT scan findings and the bronchoalveolar lavage. His general conditions completely recovered after only 24 h of oxygen supplementation and after intravenous corticosteroid and antibiotic therapy. This is the first pediatric case of DAH related to smoking marijuana, even though the inhalational anesthetic agent sevoflurane might have also been involved in this pathogenesis. Other possible causes of DAH have been considered. Negative-pressure pulmonary edema could be ruled out because no clinical evidence of upper airway obstruction was observed during general anesthesia and throughout the surgery. In addition, a possible causative role of cannabis additives/contaminants could not be excluded. Given the high prevalence of cannabis smoking in young people and that DAH can be a complication in cannabis smokers, a careful history and high index of suspicion are recommended as part of the pre-operative assessment before these patients are proceeded to receive general anesthesia.

## Background

Diffuse alveolar hemorrhage (DAH) is a medical condition characterized by bleeding into the alveolar spaces of the lungs due to the disruption of the alveolar-capillary basement membrane. It manifests as rapid onset of dyspnea, hemoptysis and acute respiratory failure (ARF) (1).

The etiology can vary from autoimmune systemic vasculitis to pulmonary infections, drugs, and tumors, which can lead to pulmonary capillaritis, bland pulmonary hemorrhage, or diffuse alveolar damage (2–4). In particular, a strong association between DAH and the use of recreational drugs, such as cocaine, amphetamine and crack, has been well-documented (5, 6).

Regarding pot smoking and DAH, literature is very poor since only few cases in adult patients have been described so far (7–11). Nonetheless, the prevalence of this recreational drug is increasing worldwide, including Italy where the lifetime prevalence of cannabis use is 27% among adolescents aged 16 years old (12, 13). In this regard, Italy has experienced a boom in light cannabis smoking since Law No. 242 went into effect December 2016, legalizing hemp cultivation and commercialization but paradoxically not its consumption (14).

In this scenario, it is more important than ever to raise a greater awareness about cannabis smoking and all the medical problems resulting from this habit. Here, we report an unprecedented case of DAH caused by cannabis abuse in a pediatric patient.

## Case Presentation

A 16-year old boy underwent laparoscopic surgery for varicocele. His past medical history was uneventful, except for febrile seizures during early childhood. He had no allergies, history of recurrent pneumonia or other chronic respiratory diseases. Likewise, his family history was unremarkable. Smoking habits were asked but not disclosed. Complete blood cells count, liver and kidney function tests and first level coagulation studies (platelets count, prothrombin time test, activated partial thromboplastin time, and fibrinogen) were all normal before the general anesthesia. According to the American Society of Anesthesiologists (ASA) risk classification^1^, he was a healthy patient (ASA 1).

Prior to the operation, the patient was given general anesthesia consisting of propofol (2 mg/kg) and rocuronium (0.6 mg/kg), with remifentanil (0.25 mcg/kg/min) for intubation. General anesthesia was maintained with 2% sevoflurane and remifentanil (0.25–0.125 mcg/kg/min). Sugammadex (2 mg/Kg) was administered before extubation (TOF ratio > 0.9). Both surgery and extubation were conducted without any complications and the patient had no obvious signs of upper airway obstruction, neither cough nor laryngospasm.

**Figure 1:**
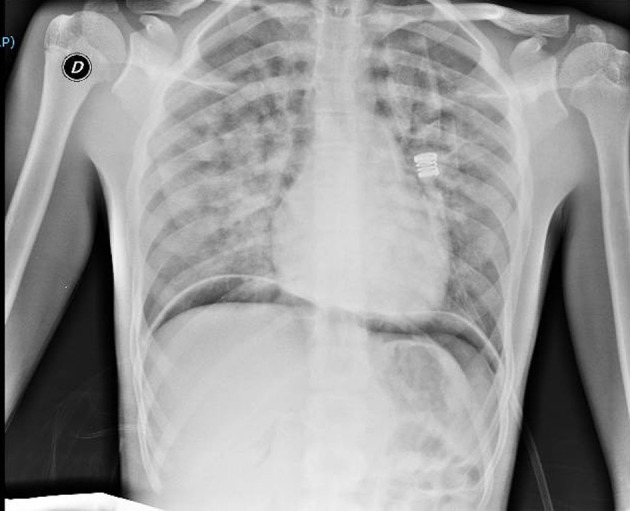
Chest x-ray at clinical onset of symptoms, consistent with diffuse alveolar hemorrhage (DAH). There can be observed infiltrative opacification pattern mainly seen in the mid zones with apical sparing suggestive for impaired pulmonary microcirculation. There can be noticed areas of subdiaphragmatic air due to laparoscopy.

**Figure 2:**
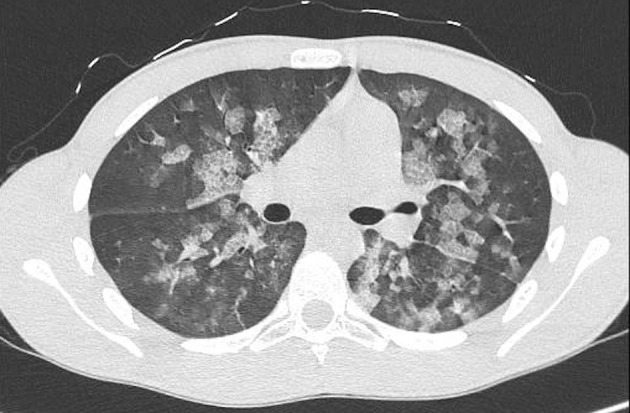
Chest CT scan without contrast medium confirming the diagnosis of DAH.

Thirty minutes after waking up, he manifested hemoptysis, dyspnea, and ARF. He was alert, tachypnoeic, and normothermic. His Glasgow Coma Scale was 15, and his initial vital signs were: pulse 80 bpm, blood pressure 90/60 mmHg and oxygen saturation averaging 73% on room air. Arterial blood gas analysis revealed: pH 7.37; pCO_2_ 39.6 mmHg (5.27 kPa); pO_2_ 39.5 mmHg (5.26 kPa); oxyhemoglobin (oxyHb) 73.7%; and HCO3^−^ 23.1 mEq/L. Otorhinolaryngoiatric evaluation with nasal and laryngeal fibro-endoscopy unveiled no evidence of previous bleeding from the upper respiratory tract. Respiratory auscultation revealed diffuse rhonchi, with no other remarkable signs. His initial chest x-ray (Figure 1) showed infiltrative opacification pattern mainly seen in the mid zones with apical sparing suggestive for impaired pulmonary microcirculation, raising the suspect of DAH (15, 16). To further investigate our hypothesis, a chest CT scan (Figure 2) was performed, which revealed multiple dense opacities with blurred, confluent margins due to diffuse alveolar involvement in the pulmonary lobes.

Given the evidence of hypoxemic respiratory failure and the suspect of DAH, the patient was transferred to the Intensive Care Unit, where he was subjected to immediate bronchoalveolar lavage, revealing the presence of abundant blood, but after the bronchoalveolar lavage with 0.9% sodium chloride solution, the bronchial mucosa appeared without signs of pathological lesions. More specifically, three lavage of 50 mLs each were done and afterwards 35, 35, and 50 mLs of BAL fluid were retrieved. Direct microscopic examination showed leucocytes, macrophages and bronchial epithelial cells. Further confirmation arrived from cytologic analysis of his bronchoalveolar lavage secretions which described the presence of blood, 85% of alveolar macrophages and 15% of granulocytes. Hemosiderin was not assessed because hemoptysis started 4 h before and the endoscopic appearance of the bleeding itself appeared to be very recent, undergoing from <48 h. The patient was given fraction inspired oxygen (FiO_2_) (40%; 12 L/min) by means of a Venturi mask for ARF, intravenous ceftriaxone 2 g *per* day as antibiotic prophylaxis and intravenous methylprednisolone 1 g *per* day for anti-inflammatory action for 5 days, while waiting for the results of microbial studies and autoimmune diseases screening (17).

Initial blood tests performed at Day 1 revealed: hemoglobin levels 13.5 g/dl (135 g/L); leukocytosis 14.540 mm^−3^ (14.54 10^9^/L), of which 83.7% were neutrophils, and normal platelet count (221 × 10^9^ /L). Renal function, liver function, electrolytes, coagulation studies (prothrombin time, activated partial thromboplastin time, and fibrinogen) were all normal. Von Willebrand factor deficiency was also ruled out on the basis of Von Willebrand factor antigen and Ristocetin cofactor activities which resulted within normal range (Von Willebrand factor antigen was 90%, with a normal reference range between 57 and 147%; Ristocetin cofactor was 120% with a normal reference range of 51–147% considering our patient's specific age group). Urinalysis was unremarkable, and direct microscopic exam did not reveal any dysmorphic red blood cells (RBCs). The diagnostic work-up, which included serological exams and blood and sputum cultures for *Streptococcus pneumoniae, Haemophilus influenzae, Legionella pneumophila, Aspergillus, Mycobacterium tuberculosis, Nocardia* spp., *Chlamydia pneumoniae, Mycoplasma pneumoniae, Pneumocystis Jirovecii, Herpesviruses*, and the complete respiratory viral panel, ruled out any pulmonary infections. An extensive rheumatological serologic evaluation including CRP, c-ANCA, p-ANCA, ANA, and ENA panel came back negative as well. Lastly, the echocardiography was completely normal, excluding any congenital or acquired cardiomyopathy as well as pulmonary hypertension.

Questioned on smoking habits, the boy revealed to be a frequent cannabis smoker since few years. He admitted to have smoked about 1–2 joints *per* day in the last month before surgery and to have used a marijuana smoking device known as “water bong” at least once. Moreover, he denied having used any other type of street drugs. We therefore performed a toxicological urine test that resulted positive for cannabinoids. Cocaine was not tested since the patient's parents declined their consent.

**Figure 3:**
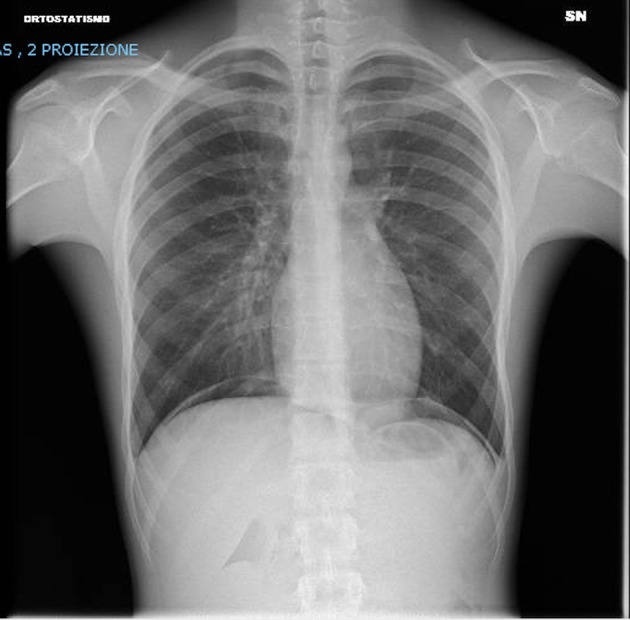
Chest x-ray after 48 h showing both lungs clear of significant parenchymal opacities and no signs of pleural effusions.

The patient's general conditions rapidly improved after 24 h: he was no longer dyspneic, and oxygen supplementation was stopped on day 2 of hospitalization after a second chest radiograph (Figure 3) revealed the resolution of DAH. He was discharged after 6 days with tapering prednisone. Although he was recommended an ambulatory follow-up, his parents declined.

## Discussion

DAH is due to disruption of the alveolar-capillary basement membrane. Common initial signs and symptoms include abrupt onset with cough, hemoptysis, fever, and dyspnea. Some patients, however, present with severe hypoxemic respiratory failure requiring immediate ventilatory support with mechanical ventilation.

Illicit drug abuse, especially cocaine, amphetamine, and synthetic cannabinoids, has been associated with DAH (5–8). To date, only five cases of DAH related to marijuana have been published, all in adult subjects. Two of these cases were individuals that had inhaled synthetic cannabinoid (7, 8), one had smoked marijuana using a homemade plastic bong (9) and died of DAH, one had smoked several marijuana joints (10) and the last one was a frequent cannabis smoker receiving general anesthesia (11). In addition, in a 16–year–old boy a case of hemoptysis was reported in association with cannabis smoking mixed with micro-particles of silicon dioxide (18). To our knowledge, our patient is the first pediatric case of DAH associated with non-synthetic cannabis smoking.

Cannabis, also known as marijuana, pot or weed, is the most popular recreational drug worldwide as it is easy to find and can be bought at an affordable price (19). According to the 2015 European School Survey Project on Alcohol and Other Drugs (ESPAD), cannabis is rated as the most widespread illicit drug in ESPAD countries, with 16% of European students having used cannabis at least once in their lifetime, ranging from 4% in Moldova to 37% in the Czech Republic. Typically, lifetime cannabis use is higher among boys than girls (19 vs. 14%, respectively) (13).

Europe is not the only geographical area with high cannabis use prevalence. A 2017 survey by the United States (US) Centers for Disease Control and Prevention (CDC) through the Youth Risk Behavior Surveillance System (YRBSS) indicates that 35.6% of US high schools students have used marijuana once or more during their life, with 6.8% of them having tried it for the first time before the age of 13 (8.3% male students; 5.3% female students) (20). Interestingly, 19.8% of US students reported current marijuana use at least once during the 30 days before taking the survey.

Even though cannabis consumption can result in generalized airway inflammation with evidence of respiratory epithelial cell injury and damage to alveolar macrophages (21), its potential role in DAH has not been well-investigated. Here, we show that DAH is associated with frequent cannabis smoking, further supporting a detrimental effect of weed smoking habit on the respiratory system of otherwise healthy individuals.

Other etiologies could explain DAH occurring after general anesthesia, such as negative-pressure pulmonary edema (NPPE). This is a well-recognized post-operative clinical entity which has been described as causative factor of DAH. Generation of a negative pleural pressure on inspiration against an upper airway obstruction is the mechanism of stress failure of the alveolar-capillary membrane in NPPE (22). In our case, we did not observe airway obstruction during general anesthesia and the surgery was conducted without any complications.

Another limitation of our study is that we cannot rule out that the anesthetic drugs used for induction and maintenance of general anesthesia could have also played a role in DAH. All of them are somehow related to pulmonary complications. For instance, sugammadex is known to cause negative-pressure pulmonary edema, remifentanil is related to respiratory depression, as well as propofol to acute lung injury and pulmonary edema^2^ Regarding rocuronium, no evidence of lung injury effect exists. In some cases sevoflurane has been associated to DAH. In the literature, 4 cases of sevoflurane-related DAH have been reported, with one of them being a cannabis smoker (11, 23–25). In this regard, it has been proposed that sevoflurane, as well as other inhalational anesthetic agents, could increase alveolar permeability, oxidative stress and inflammatory response, thereby promoting DAH. Moreover, in one case report, a young adult patient developed DAH due to negative-pressure pulmonary edema as a result of muscle rigidity after a bolus of remifentanil. This was reversed by neuromuscular blockade with sugammadex (26). Thus, it is not only difficult to disentangle the possible additive or synergistic effects of anesthetic drugs and cannabis smoke, but it is also tempting to speculate that the chronic cannabis use may have predisposed our patient to airway inflammation and alveolar epithelial damage, which could have been exacerbated by sevoflurane inhalation, resulting in inhalational injury.

Our speculation is limited by a paucity of information about our patient's smoking habits. We did not know how long our patient had been smoking and the declared amount of joints per day may be underestimated. Moreover, we did not know how much cannabis and tobacco were used to prepare joints. For instance, psychoactive inert herbs or synthetical additives may be mixed to Cannabis. Even if a causative role of cannabis additives/ contaminants in airways inflammation could not be excluded, our patient declared to smoke only *Cannabis sativa* and anatomopathological analysis of his bronchoalveolar lavage secretions did not describe the presence of any additives or contaminants.

In addition to that, cocaine use should be considered in the differential diagnosis of DAH. However, we did not know if our patient ever used cocaine since he denied this habit and his parents declined their consent to the specific test.

Another limitation is the incomplete and perhaps unreliable patient history, combined with lack of any information regarding the follow-up due to the refusal of the patient's parents to collaborate. The follow-up would possibly have corroborate our speculation, because it is unlikely that smoking joints had been stopped and, if there actually was a close link to the DAH, it might have relapsed.

## Conclusions

Although it is still not clear how high the risk of cannabis-related DAH may be, the high prevalence of cannabis smoking among young adolescents calls for a more thorough assessment of this potential risk before any surgery under general anesthesia. In this regard, further studies are urgently needed to confirm the association between marijuana smoking and lung injury.

Therefore, despite the absence of a precise estimation of the risk, we recommend caution in cannabis smokers undergoing general anesthesia, as they may be more susceptible to develop respiratory complications, such as DAH.

## Author Contributions

LB and AM contributed conception and design of the study. LB wrote the first draft of the manuscript. SF and TC wrote sections of the manuscript. AM, GG, and SB reviewed and edited the manuscript. All authors contributed to manuscript revision, read, and approved the submitted version.

### Conflict of Interest

The authors declare that the research was conducted in the absence of any commercial or financial relationships that could be construed as a potential conflict of interest.

## Footnotes

^1^https://www.asahq.org/standards-and-guidelines/asa-physical-status-classification-system

^2^https://www.pneumotox.com/drug/index/

## Abbreviations

ARF: acute respiratory failure
DAH: diffuse alveolar hemorrhage.
